# Photoluminescence lifetime engineering via organic resonant films with molecular aggregates

**DOI:** 10.1515/nanoph-2023-0631

**Published:** 2024-01-08

**Authors:** Kyu-Ri Choi, Shilong Li, Dong Hee Park, Bin Chan Joo, Hojun Lee, Evan S. H. Kang, Síle Nic Chormaic, Jeong Weon Wu, Anthony D’Aléo, Yeon Ui Lee

**Affiliations:** Chungbuk National University, Cheongju, Republic of Korea; Okinawa Institure of Science and Technology Graduate University, Onna, Japan; Ewha Womans University, Seoul, Republic of Korea; Université de Strasbourg, Strasbourg, France

**Keywords:** lifetime engineering, Purcell effect, organic ENZ/ENP materials

## Abstract

Manipulating the spontaneous emission rate of fluorophores is vital in creating bright incoherent illumination for optical sensing and imaging, as well as fast single-photon sources for quantum technology applications. This can be done via increasing the Purcell effect by using non-monolithic optical nanocavities; however, achieving the desired performance is challenging due to difficulties in fabrication, precise positioning, and frequency tuning of cavity-emitter coupling. Here, we demonstrate a simple approach to achieve a wavelength-dependent photoluminescence (PL) lifetime modification using monolithic organic molecular aggregates films. These single monolithic organic films are designed to have a Lorentzian dispersion, including epsilon-near-zero (ENZ) and epsilon-near-pole (ENP) spectral regions with increased and decreased photonic density of states, respectively. This dispersion leads to enhanced and depressed PL decay rates at different wavelengths. Both time-resolved photoluminescence (TRPL) and fluorescence lifetime imaging microscopy (FLIM) measurements are implemented to verify the validity of this approach. This approach offers a promising way to design dual-functional optical sources for a variety of applications, including bioimaging, sensing, data communications, and quantum photonics applications.

## 1 Introduction

Spontaneous emission (SE) manipulation of fluorophores has been of a great interest in many optical applications, such as bioimaging [1], [2], [3], displays [4], and optoelectronic devices [5], [6]. One of the possible approaches that can control the SE decay rate of the fluorophores is the modification of the photonic local density of state (PLDOS) in the nearby optical medium, described as the Purcell effect. The PLDOS refers to the number of photonic states per energy in the given optical medium where photons are around. Therefore, PLDOS is a parameter controlling the SE process which is determined by the nearby optical medium (Supporting Information I). Over the past two decades, substantial progress has been made in developing various optical media, such as photonic crystals [7], plasmonic metamaterials [8], [9], and whispering-gallery-mode microcavities [10], to efficiently manipulate the PLDOS and thus the SE decay rate of the fluorophores. Despite the potential benefits of these optical media in various applications, they are faced with several challenges. Firstly, their high-cost and complex fabrication processes hinder their widespread use. Secondly, a precise positioning is often required but challenging to achieve, presenting a significant obstacle [11]. Last but not least, tuning them to operate at specific wavelengths can be difficult, leading to a narrow operational bandwidth.

In recent developments, it has become possible to modify the PLDOS by placing fluorophores onto spin-coated monolithic organic films, without the need for the precise positioning. For example, organic thin films with a layered molecular packing structure, such as quinoidal oligothiophene derivative (QQT(CN)4) [12], and regioregular poly(3-hexylthiophene-2,5-diyl) (rr-P3HT) [13], [14], have been utilized to enhance the PLDOS by up to two orders of magnitude [13]. These films support high-spatial frequency (high-*k*) optical modes in the spectral range of hyperbolic dispersion which contribute to the enhancement of the PLDOS. The hyperbolic dispersion is a result of an anisotropic molecular aggregates structure, which is usually accompanied by several intriguing spectral regions, including the epsilon-near-zero (ENZ) and epsilon-near-pole (ENP) regions. However, the wavelength-dependent fluorescence lifetime – particularly the demonstration of both lengthening and shortening of the fluorescence lifetime at different spectral regions – has yet to be studied.

In this article, we demonstrate a wavelength-dependent fluorescence lifetime modification with an organic ENZ/ENP thin film. The ENZ/ENP thin film [15], [16], [17] used is the 2,4-bis[8-hydroxy-1,1,7,7-tetramethyljulolidin-9-yl]squaraine (HTJSq), while the fluorescence is from the rr-P3HT molecules. Time-resolved photoluminescence (TRPL) measurements are carried out to investigate the fluorescence dynamics of the rr-P3HT molecules on top of the HTJSq film. Purcell factor calculations and fluorescence lifetime imaging microscopy (FLIM) [18], [19] are utilized to verify the validity of this fluorescence-engineering approach.

## 2 Results and discussion

**Figure 1:**
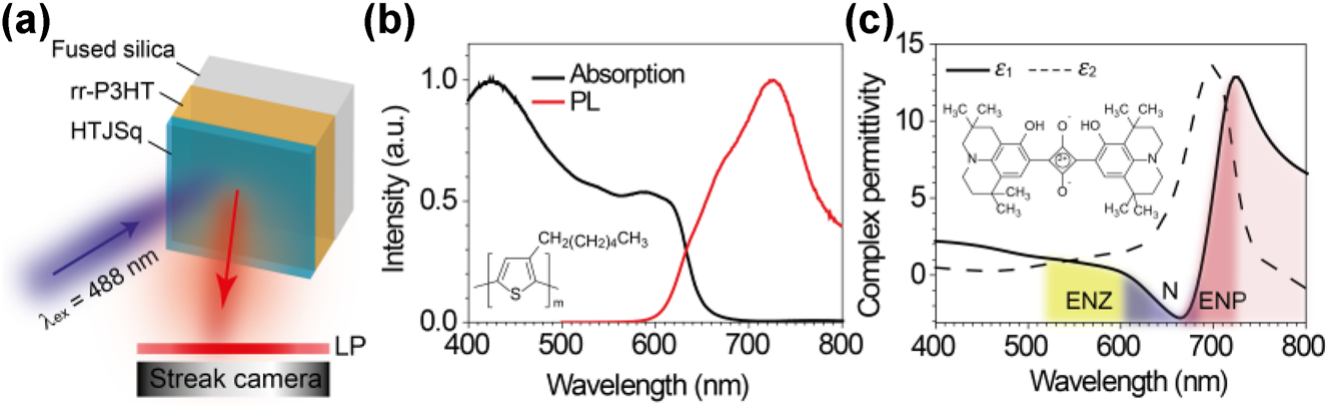
Optical responces of individual organic films. (a) Schematic illustration of the TRPL measurement. A femtosecond laser at the excitation wavelength of 480 nm is incident on the sample. The emission from the sample is filtered by a 600 nm long-pass (LP) filter before being detected by a streak camera. (b) Measured absorption and PL spectra of rr-P3HT film. (c) Measured complex dielectric permittivity of the HTJSq film.

The experimental setup is schematically shown in Figure 1a. The sample used in our experiments is a bi-layer film consisting of spin-coated rr-P3HT and thermally evaporated HTJSq (Supporting Information II). 120-mg/mL rr-P3HT in chlorobenzene solution was spin-coated at 5000 rpm for 60 s. HJTSq film was thermally evaporated on top of rr-P3HT film with an average evaporation rate 0.5 Å/s at vacuum of 1.0 × 10–6 Torr. The PL spectrum of the rr-P3HT film is given in Figure 1b. The rr-P3HT has a broad emission band centered at approximately 730 nm and was used as the fluorophore. The HTJSq film was optimized to have a Lorentzian dispersion with both ENZ (530–610 nm) and ENP (691–725 nm) regions, as well as a negative epsilon (N) region around 610–691 nm (Figure 1c) between them. The presence of the HTJSq film modifies the fluorescence dynamics of the underneath rr-P3HT in a wavelength-dependent manner. To study these dynamics time-resolved photoluminescence (TRPL) system (Supporting Information III) was employed, and the results are discussed below.

**Figure 2:**
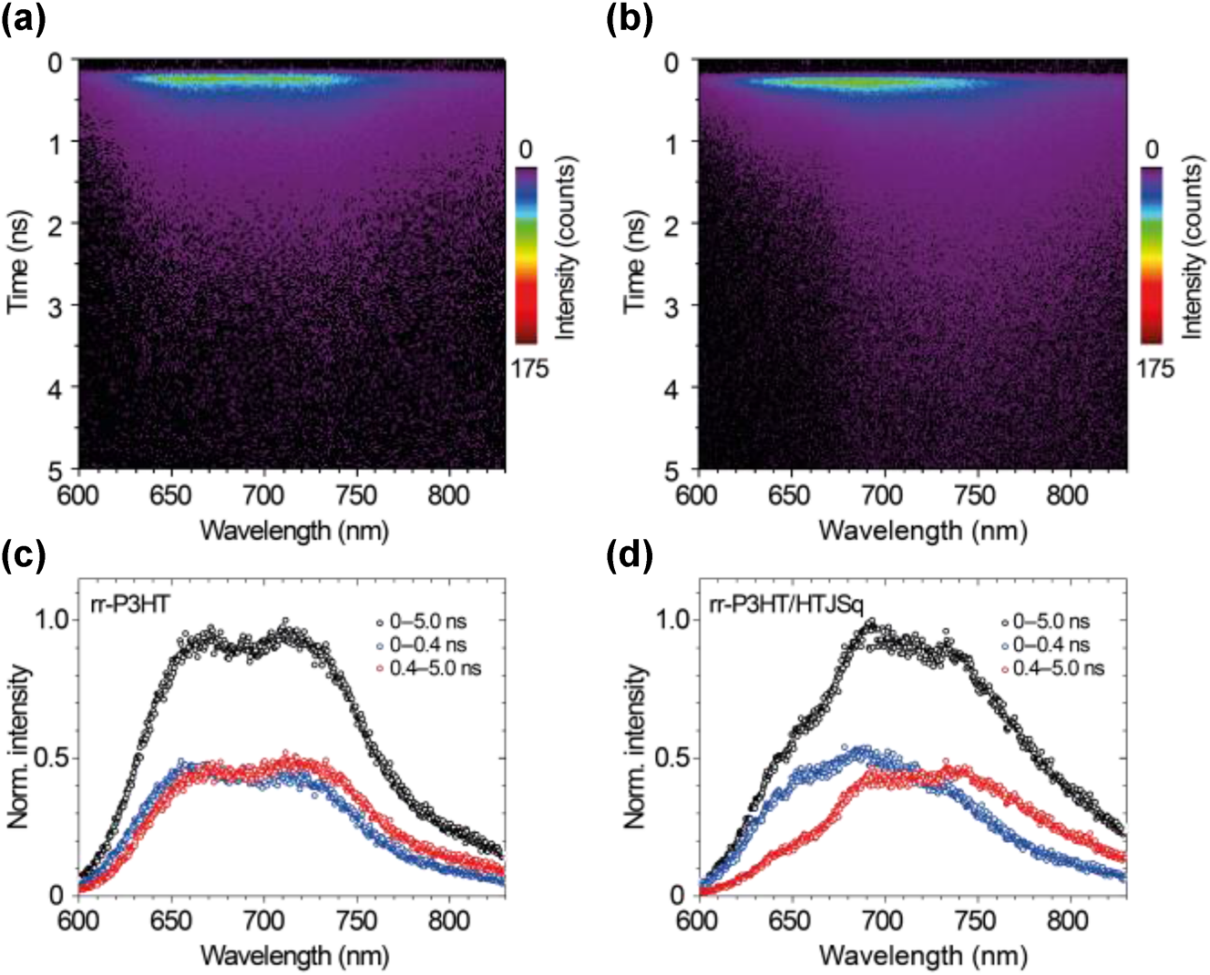
TRPL spectra of (a) the bare rr-P3HT film and (b) the rr-P3HT film covered by HTJSq. The corresponding integrated PL intensity spectra for different time intervals are given in (c) and (d).

Figure 2a and b summarize the TRPL results for rr-P3HT films with and without the HTJSq top layer, respectively. There is a noticeable change in the fluorescence dynamics of the rr-P3HT when coated with a 5-nm thin layer of HTJSq. This observation confirms that it is an easy and efficient approach for fluorescence engineering. To further clarify the wavelength dependence of this approach, the integrated PL intensity specifically in the ENP region. In contrast, the slow decay process experiences a decrease in intensity in the ENZ + N region. These findings provide new insight into the effects of HTJSq on the wavelength-dependent fluorescence dynamics in different decay processes.

**Figure 3:**
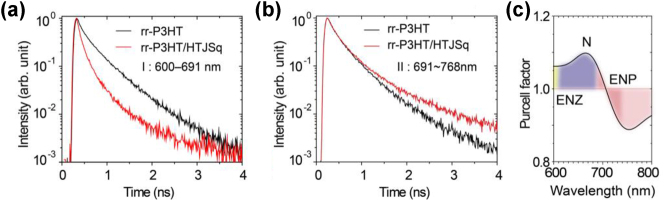
Spectrally resolved PL decay curves in the spectral ranges of (a) 600–691 nm and (b) 691–768 nm. (c) Calculated wavelength-dependent Purcell factor.

The wavelength dependence of the fluorescence dynamics of the rr-P3HT can also be visualized in the spectrally resolved PL decay curves, as shown in Figure 3. Two spectral ranges, i.e. 600–691 nm and 691–768 nm, were examined based on the analysis above, corresponding to the ENZ + N and ENP regions, respectively. In the ENZ + N spectral region, the HTJSq coated rr-P3HT shows an accelerated PL decay of 47 % compared to the rr-P3HT film (Figure 3a, see also Table S1 in Supporting Information IV). It is related to the slow light effect resulting from exciton polariton modes near the ENZ + N [8], [20] spectral region, which alters any light–matter interaction processes including the spontaneous decay. It is interesting to note that we observe not only an accelerated PL decay but also a decelerated PL decay of 9 % in the rr-P3HT/HTJSq film in the ENP spectral region (Figure 3b, see also Table S1 in Supporting Information IV). It is the result of the modification of PLDOS in the ENZ + N and ENP regions which is provided by the resonant absorption of the HTJSq molecule aggregation. Nevertheless, the Purcell factor [21] is the most relevant physical quality to measure the change of the spontaneous decay process when the local photonic environment is varied [22]; here, the local photonic environment is determined by the HTJSq thin film via its nontrivial dielectric permittivity (Figure 1c). Therefore, FDTD simulations (Supporting Information III) were conducted to obtain the Purcell factor of the rr-P3HT/HTJSq film system and the results are summarized in Figure 3c. It clearly shows that the Purcell factor is enhanced (depressed) in the ENZ + N (ENP) region which is attributed to the accelerated (decelerated) spontaneous decay of the rr-P3HT/HTJSq film. A detailed discussion on the physical mechanism of PLDOS, spontaneous emission lifetime, and Purcell factor is given in Supporting Information I.

**Figure 4:**
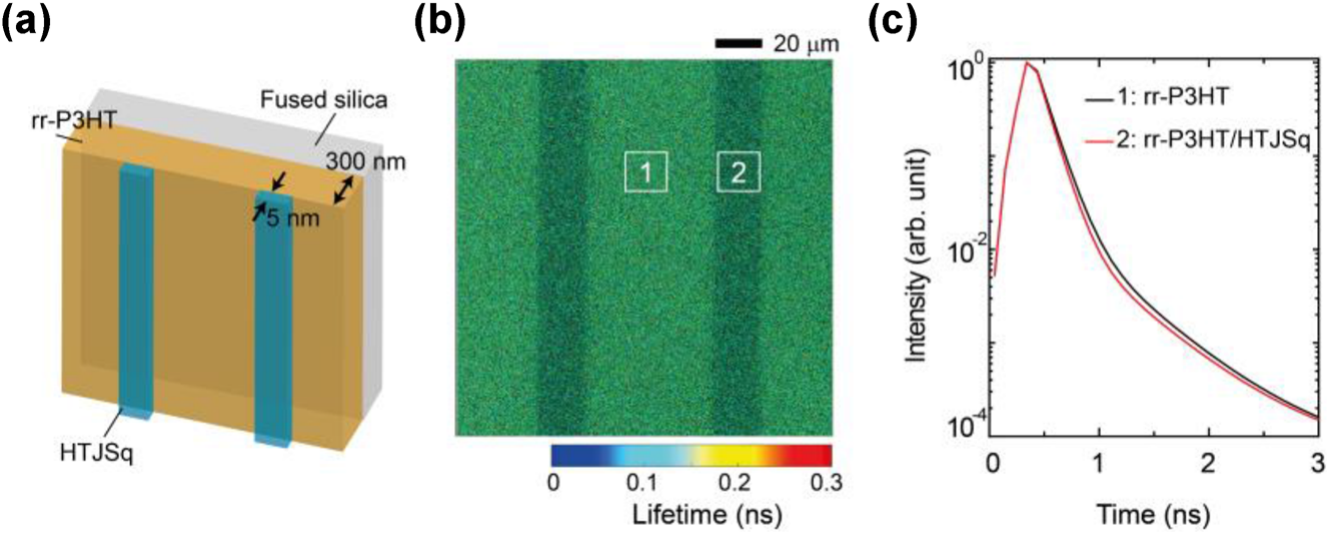
FLIM results for the organic bi-layer film. (a) Sample structure used in FLIM experiments. (b) FLIM image of the sample. (c) FLIM decay curves at different sample positions indicated in (b).

It is worth noting that the spontaneous lifetime modification by HTJSq thin films is a near-field effect: Only those rr-P3HT fluorescent molecules near the HTJSq films are affected. As a result, there are at least two decay processes associated with the affected and unaffected rr-P3HT fluorescent molecules, which leads to a multi-exponential decay curve in our experimental condition as shown in Figures 3 and 4 (see details about the decay curve fitting in Supporting Information IV).

To further verify the validity of fluorescence lifetime engineering with HTJSq thin films, fluorescence lifetime imaging microscopy (FLIM) experiments were carried out (Supporting Information III). To have a straightforward comparison, the sample was fabricated to consist of periodically structured HTJSq layers on top of the rr-P3HT film, as shown in Figure 4a. In the FLIM experiment, the excitation wavelength was set at 480 nm, while the PL signal was detected after a band pass filter of 600–680 nm, which corresponds to the ENZ + N spectrum region of the HTJSq layer, with FLIM image shown in Figure 4b. It shows that the PL lifetime of the rr-P3HT is shorter in the presence of the HTJSq top layer, corresponding to an accelerated PL decay following the tendency of the PL measurements, as shown in Figure 4c. These FLIM results are consistent with those obtained in the TRPL experiments, as discussed above.

In summary, we have demonstrated a simple approach to realize a wavelength-dependent photoluminescence lifetime modification by using an organic ENZ/ENP thin film in the visible spectral range. Both FLIM and TRPL experiments were carried out to confirm the validity of this approach. Compared to the plasmonic cavities photoluminescence lifetime modification with the flat thin film has little need for the alignment, and it has a broad-spectrum response compared to the dielectric cavities with a high qualify factor. The photoluminescence intensity after the ENZ/ENP thin film coating drops due to the introduced nonradiative processes; patterning the thin film into nanoantenna structures could help to improve the photoluminescence intensity. Since the organic ENZ/ENP materials used are nontoxic and biocompatible, the demonstrated approach enables new possibilities in the development of optical material platforms for biosensing and bioimaging.

## Supplementary Material

Supplementary Material Details
