# Supplementary material for: Photoluminescence lifetime engineering via organic resonant films with molecular aggregates
Source: Nanophotonics. 2024 Jan 8;13(7):1033–7. doi: 10.1515/nanoph-2023-0631 (PMC11501593; doi:10.1515/nanoph-2023-0631)
Supplement: Supplementary file 1 — Supplementary Material Details [file j_nanoph-2023-0631_suppl_001.docx]

Supporting Information

**Photoluminescence lifetime engineering via organic resonant films with molecular aggregates**

Kyu-Ri Choi^1^, Shilong Li^2^, Dong Hee Park^1^, Bin Chan Joo^1^, Hojun Lee^1^, Evan S. H. Kang^1^, Síle Nic Chormaic^2^, Jeong Weon Wu^3^, Anthony D'Aléo^4,^*, and Yeon Ui Lee^1,^*

^1^Department of Physics, Chungbuk National University, Cheongju, Chungbuk 28644, Republic of Korea

^2^Light-Matter Interactions for Quantum Technologies Unit, Okinawa Institute of Science and Technology Graduate University, Onna, Okinawa 904-0495, Japan

^3^Department of Physics, Ewha Womans University, Seoul 03760, Republic of Korea

^4^CNRS-IPCMS (UMR 7504), Université de Strasbourg, Strasbourg 67034, France

*Corresponding author. Email: [anthony.daleo@ipcms.unistra.fr](mailto:anthony.daleo@ipcms.unistra.fr), [yeonuilee@cbnu.ac.kr](mailto:yeonuilee@cbnu.ac.kr)

**Contents**

**I. Discussion on PLDOS and spontaneous emission lifetime**

**II. Sample preparation**

**III. Methods**

**IV. TRPL lifetime fitting result**

**I. Discussion on PLDOS and spontaneous emission lifetime**

Applying the Fermi’s golden rule for the spontaneous emission process in a two-level system, one can obtain the spontaneous emission rate of electron in the excited state in vacuum:

$\Gamma_{\mathrm{SpE}}=\frac{\mu^{2}\omega^{3}}{3\pi c^{3}\hbar\varepsilon_{0}}\equiv\frac{1}{\tau_{R}}$, (S1)

where $\mu$ is the transition dipole moment, $\omega$ is the emission frequency, $c$ is the vacuum speed of light, $\hbar$ is the reduced Planck constant, $\varepsilon_{0}$ is the vacuum permittivity, and $\tau_{R}$ is the spontaneous emission lifetime. To get $\Gamma_{\mathrm{SpE}}$, the photonic vacuum density of state $\rho_{\mathrm{BB}}$ is used:

$\rho_{\mathrm{BB}}=\frac{2\omega^{2}}{\pi c^{3}}$. (S2)

When the two-level system is placed near a photonic structure, the spontaneous emission rate is modified according to the photonic local density of state (PLDOS) $\rho_{c}$, which can be well measured by Purcell factor:

$F_{P}\equiv\frac{\Gamma_{\mathrm{SpE},c}}{\Gamma_{SpE,BB}}\equiv\frac{\tau_{R,BB}}{\tau_{R,c}}=\frac{\rho_{c}}{\rho_{\mathrm{BB}}}$. (S3)

For a photonic cavity made of dielectric materials, one has:

$F_{P}=\frac{3}{4\pi^{2}}\frac{Q}{V}\lambda^{3}$, (S4)

while it is:

$F_{P}=\frac{3}{4\pi^{2}}\mathrm{Re}(\frac{Q}{V})\lambda^{3}\sim\frac{1}{V^{2}}$, (S5)

for a plasmonic cavity. Here, $Q$ is the quality of a mode, $V$ is the mode volume, and $\lambda$ is the mode wavelength. As for a film with ENZ or ENP spectral region, there is no analytical solution for the Purcell factor $F_{P}$; therefore, various simulation methods, such as FDTD, are used.

**II. Sample preparation**

All reagents were purchased from Sigma Aldrich or TCI and were used as supplied without further purification. 120 mg/mL rr-P3HT in chlorobenzene solvent was heated at 70 °C for 3 hours. Substrates were cleaned in acetone, isopropyl alcohol (IPA), and chloroform, with excess liquid air blown. The P3HT solution was spin-coated on top of substrates at 5000 rpm for 60 s. HJTSq film was thermally evaporated on top of rr-P3HT film with an average evaporation rate 0.5 Å/s at vacuum of 1.0×10^–6^ Torr.

**III. Methods**

**Spectroscopic ellipsometry:** A spectroscopy ellipsometer (J. A. Woollam Co. alpha-SE) was used to measure Ψ and Δ in the wavelength range of 381–887 nm at the angle of incidence of 70°. These are used to describe the output polarization state after linearly polarized light is reflected from a thin film. Note that the light beam illuminating the samples had a diameter of ∼2 mm. After measuring Ψ and Δ, a model was built using the analysis software CompleteEASE® to determine the optical constants and the dielectric permittivity of the film.

**Time resolved photoluminescence measurements:** A Ti-sapphire pulsed laser (Spectra-Physics) was used to deliver >3.5-mJ pulses at 800 nm with a pulse width of ~35 fs at a repetition rate of 5 kHz. Pulses at 480 nm were obtained via second harmonic generation of 960 nm with a BBO crystal with a spot size diameter of ~1.5 mm. Optical parametric amplifier (OPA) provides pulses with various wavelengths through optical parametric amplification processes.

**Finite-difference time-domain (FDTD) simulations:** FDTD simulations (Ansys Lumerical) were carried out to calculate the Purcell factor. The film thickness of the organic thin films obtained through surface profilometry (Dektak) measurements were used along with the permittivity values obtained through the spectroscopic ellipsometry in these simulations. Purcell factor calculation was carried out by placing a dipole source on top of HTJSq film, the power emitted from the dipole in the presence of HTJSq divided by the power emitted from the dipole in the absence of HTJSq is calculated upon a 600–800 nm. The geometries of the sample structure in the simulations were designed to match with those displayed in Figure 1a. Mesh size was kept at 40 nm for all three axes with an override mesh size for the HTJSq film of 2 nm. The FDTD simulation dimension span was 1000×1000 nm^2^ for the x-y plane and 3000 nm for the z-axis with PML boundary conditions.

**Fluorescence lifetime imaging microscopy (FLIM):** Time-resolved fluorescence lifetime study was conducted using an inverted-type scanning confocal microscope (SP8 FALCON, Leica Microsystems, Germany) with a 40× (air) objective lens. The fluorescence lifetime measurement was conducted at KBSI, Daegu Center, Korea. A picosecond pulsed laser (480 nm, 40 MHz) was used as the excitation source. A hybrid photon detector was used to collect emissions in the range of 600–680 nm from the sample. Confocal and fluorescence lifetime images of 512×512 pixels were simultaneously recorded using a galvo-stage and time-correlated single-photon counting technique.

**IV. TRPL lifetime fitting result**

According to the FDTD simulations, the lifetime modification by the ENZ/ENP HTJSq film is a near-field effect, that is, only those rr-P3HT fluorescent molecules near the HTJSq film are affected. As a result, there are at least two decay processes associated with the affected and unaffected rr-P3HT fluorescent molecules, which leads to a multi-exponential decay curve in our experimental condition. To take into account these decay processes, the measured TRPL curves were fitted with three exponential functions $I\left( t \right)=A_{1}e^{-t/\tau_{1}}+A_{2}e^{-t/\tau_{2}}+A_{3}e^{-t/\tau_{3}}+C$, and the fitting result is summarized in Table S1. The average lifetime $\tau_{f}$, the radiative decay rate $k_{f}$, and the non-radiative decay rate $k_{nr}$ are also listed.

**Table S1:** TRPL lifetime fitting result.

|  | Region | $\tau_{1}$ (ps) | $\tau_{2}$ (ps) | $\tau_{3}$ (ps) | $A_{1}$ | $A_{2}$ | $A_{3}$ | $\tau_{f}$ (ps) | $k_{f}$ (μs^-1^) | $k_{nr}$ (μs^-1^) |
| --- | --- | --- | --- | --- | --- | --- | --- | --- | --- | --- |
| rr-P3HT | I | 152 | 509 | 1864 | 0.642 | 0.349 | 0.001 | 277 | 667 | 2943 |
|  | II | 175 | 483 | 1917 | 0.537 | 0.450 | 0.014 | 338 | 548 | 2412 |
| rr-P3HT/HTJSq | I | 98 | 345 | 1823 | 0.798 | 0.195 | 0.001 | 147 | 1237 | 5566 |
|  | II | 183 | 526 | 1892 | 0.606 | 0.357 | 0.037 | 369 | 494 | 2219 |
